# Reduced Alcohol Seeking and Withdrawal Symptoms in Mice Lacking the BDNF Receptor SorCS2

**DOI:** 10.3389/fphar.2019.00499

**Published:** 2019-05-17

**Authors:** Ditte Olsen, Mathias Kaas, Jesper Lundhede, Simon Molgaard, Anders Nykjær, Mads Kjolby, Søren D. Østergaard, Simon Glerup

**Affiliations:** ^1^Department of Biomedicine, Aarhus University, Aarhus, Denmark; ^2^Department of Biomedicine, Nordic EMBL Partnership for Molecular Medicine, DANDRITE – Danish Research Institute of Translational Neuroscience, Aarhus University, Aarhus, Denmark; ^3^Department of Neurosurgery, Aarhus University Hospital, Aarhus, Denmark; ^4^The Danish National Research Foundation Center PROMEMO, Aarhus University, Aarhus, Denmark; ^5^Danish Diabetes Academy, Novo Nordisk Foundation, Aarhus, Denmark; ^6^Department of Clinical Medicine, Aarhus University Hospital, Aarhus, Denmark; ^7^Department of Affective Disorders, Aarhus University Hospital – Psychiatry, Aarhus, Denmark; ^8^The Lundbeck Foundation Initiative for Integrative Psychiatric Research, iPSYCH, Aarhus, Denmark

**Keywords:** SorCS2, alcohol, alcohol use disorder, alcohol withdrawal, BDNF and VPS10p-domain receptor

## Abstract

Alcohol use disorder (AUD) is characterized by repetitive and uncontrolled intake of alcohol with severe consequences for affected individuals, their families and society as a whole. Numerous studies have implicated brain-derived neurotrophic factor (BDNF) activity in the neurobiology underlying AUD. The BDNF signaling mechanism is complex and depends on two receptor systems, TrkB and p75NTR, which appear to have opposite effects on alcohol seeking behavior in animal models. We recently discovered that the sortilin-related receptor SorCS2 forms complexes with both TrkB and p75NTR and is important for BDNF activity in the developing and adult CNS. Moreover, the *SORCS2* gene was recently identified as the top association signal for severity of alcohol withdrawal symptoms. Hence, we speculated that SorCS2 deficient mice would have an altered response to alcohol. The role of SorCS2 in the acute and adapted response to alcohol was therefore investigated by comparing SorCS2 knockout (*Sorcs2^−/−^*) mice to wild type (WT) mice in three paradigms modeling alcohol sensitivity and consumption; alcohol-induced conditioned place preference, two-bottle choice test as well as the behavioral response to alcohol withdrawal. We found that, when compared to the WT mice, (I) *Sorcs2^−/−^* mice displayed complete lack of alcohol-induced place preference, (II) when given free choice between water and alcohol, *Sorcs2^−/−^* mice consumed less alcohol, and (III) *Sorcs2^−/−^* mice showed no handling-induced convulsion in response to alcohol withdrawal following extended alcohol exposure. Taken together, these results show that lack of the alcohol withdrawal risk gene *Sorcs2* results in abnormal behavioral response to alcohol in mice. Consequently, SorCS2 may play an important role in the molecular pathways underlying AUD and complications associated with alcohol withdrawal.

## Introduction

Alcohol is widely consumed throughout the world and is socially accepted in many societies. Unfortunately, approximately 12% of consumers develop alcohol use disorder (AUD) and 4% become addicted to alcohol (WHO, 2014). Approximately 3.3 million deaths a year and 5% of the global burden of disease is attributable to harmful alcohol consumption (WHO, 2014).

Alcohol use disorder is associated with altered function and plasticity of multiple neuronal systems, in particular involving impaired N-methyl-D-aspartate (NMDA) receptor and dopaminergic activity. These alterations affect the acute sensitivity to alcohol, and the development of tolerance to and dependence upon alcohol (Ron and Barak, 2016). Brain-derived neurotrophic factor (BDNF) is a key regulator of neuronal development and plasticity, and has repeatedly been implicated in the neurobiology of addiction – including AUD (Logrip et al., 2015; Koskela et al., 2017). The neurotrophic effects of BDNF on neuronal survival, differentiation, synapse formation and synaptic long-term potentiation is mediated by the receptor tyrosine kinase TrkB (Park and Poo, 2013). On the other hand, BDNF and its proform proBDNF signal cell death, growth cone collapse and synaptic long-term depression through the TNF-alpha-receptor homolog p75NTR (Deinhardt et al., 2011; Glerup et al., 2014b, 2016). Thus, lack of one of these components sometimes show opposite effects on neuronal systems compared to lack of the other component, as exemplified by the behavioral phenotypes of mice deficient for either BDNF, TrkB, or p75NTR, which represents opposite poles of the spectrum with relation to anxiety and fear memory (Olsen et al., 2013). The counteracting effect of p75NTR on TrkB activity is also illustrated in the dorsolateral striatum in the regulation of alcohol seeking behavior in rats. Here, BDNF signaling through TrkB initially suppresses alcohol intake during voluntary alcohol consumption (Jeanblanc et al., 2009). However, long-term excessive alcohol intake induces an upregulation of p75NTR thereby inducing a change in the outcome of BDNF signaling by counteracting TrkB activity and eliminating its suppressive effects (Darcq et al., 2016).

We recently found that SorCS2 from the Vps10p (vacuolar protein sorting 10 protein) domain receptor family (Glerup et al., 2014a), forms complexes with both p75NTR (Glerup et al., 2014b), and TrkB (Glerup et al., 2016). Activation of the SorCS2/p75NTR complex by proBDNF is critical for growth cone collapse and guidance of midbrain dopaminergic axons during development (Glerup et al., 2014b) and for the induction of NMDA receptor-dependent long-term depression (LTD) in the hippocampus (Glerup et al., 2016). Conversely, SorCS2 complex formation with TrkB is essential for BDNF-induced hippocampal long-term potentiation (LTP) and for BDNF-induced changes in dendritic complexity and spine density (Glerup et al., 2016). *Sorcs2^−/−^* mice display increased dopaminergic innervation of the frontal cortex albeit reduced dopamine levels and increased dopamine turnover (Glerup et al., 2014b). Thus, three systems, BDNF, NMDA receptor, and dopaminergic activity, that are considered main culprits in AUD (Ron and Barak, 2016), are all perturbed in *Sorcs2^−/−^* mice. Moreover, SORCS2 has been genetically associated with the severity of alcohol withdrawal in alcohol dependent individuals (Smith et al., 2018). We therefore speculated whether *Sorcs2^−/−^* mice would have altered response to alcohol compared to WT mice – and tested this using three paradigms modeling alcohol consumption and withdrawal: (I) alcohol-induced conditioned place preference, (II) the two-bottle choice test, and (III) Handling-induced withdrawal convulsions (HIC) after inducing alcohol dependency.

## Materials and Methods

### Animal Experiments

All experiments were approved by the Danish Animal Experiments Inspectorate under the Ministry of Justice (Permit 2011/561-119, 2016-15-0201-01127 and 2017-15-0201-01192) and carried out according to institutional and national guidelines. All animals were bred and housed at the Animal Facility at Aarhus University. *Sorcs2^−/−^* mice had been backcrossed for ten generations into C57/BL6J Bomtac background (Glerup et al., 2014b). Animals were housed in groups of up to six mice per plastic cage (42 × 25 × 15 cm) with a 12-h light/12-h dark schedule (light on 6.00 a.m. and light off at 6.00 p.m.) and fed standard chow (Altromin #1324) and water *ad libitum*. Cages were cleaned every week and supplied with bedding and nesting material, a wooden stick, and a metal tunnel. Behavioral experiments were carried out using adult age-matched male mice (between 13 and 18 weeks, weighing between 24 and 35 g) during their light cycle between 9:00 a.m. and 5:00 p.m. Each of the behavioral tests described below were carried out using naïve animals. All experiments were conducted twice independently with the exception of the two-bottle choice test. At the end of behavioral experiments, animals were sacrificed by cervical dislocation.

### Alcohol-Induced Place Preference

Conditioned place preference (CPP) is a classical Pavlovian conditioning where a stimulus, in this case alcohol, is coupled to a particular place (Tzschentke, 2007). The experiment was conducted using a place preference box (Harvard Apparatus), which consisted of two larger conditioning compartments on either side of a smaller connecting compartment. The two conditioning compartments differed in color on the walls and floor (black/white), texture of the floor (rippled/normal flat), and illumination (dim/bright). The connecting compartment was neutrally gray with intermediate illumination. The CPP protocol was modified from de Carvalho et al. (2010) and consisted of an 8-day schedule divided into three phases: habituation (1 day), conditioning (6 days), and post-conditioning (1 day). In the habituation phase, the mice were allowed to roam freely in all three compartments for 20 min per day. The time spent in each of the three compartments was recorded. Mice displaying unconditioned preference (≥85% of the time spent in a particular compartment) or aversion (≤15% of the time spent in a particular compartment) were excluded from the remaining procedure. During the 6 days conditioning phase, the mice (WT *n* = 6 and *Sorcs2^−/−^ n* = 10) were confined to one of the lateral compartments for 15 min immediately after intraperitoneal administration of ethanol (1 g ethanol/kg bodyweight) and, on alternate days, confined to the opposite compartment after administration of vehicle. In the post-conditioning phase, the mice were placed in the neutral compartment with free access to the two lateral compartments for 20 min allowing assessment of CPP. The mice were recorded with a camera in the ceiling and tracked and analyzed using the ANY-maze tracking software (Stoelting). The preference index was calculated as the ratio of time in the EtOH-conditioned chamber compared to the saline-conditioned chamber. A mixed ANOVA was used to analyze CPP data with genotype and time (time spent in EtOH-conditioned compartment/time spent in saline-conditioned chamber) as factors. A mixed ANOVA was used to analyze activity data with genotype and activity as factors. *Post hoc* analysis was performed with Sidak’s multiple comparison test.

### Contextual and Cue-Dependent Fear Conditioning

To rule out that the lack of place preference in *Sorcs2^−/−^* mice was due to deficient contextual memory, we performed a context- and cue-dependent fear conditioning experiment, which assesses hippocampus- and amygdala-dependent memory. Contextual and cue-dependent fear conditioning was performed essentially as described in Chen et al. (2006) using the StartFear system from Panlab. The mouse shock-chamber was set up in a sound attenuated box and initially scented with peppermint odor (0.1%). On day 1, the mice (WT *n* = 21 and *Sorcs2^−/−^ n* = 18) were allowed to explore the chamber for 2 min before the presentation of three cycles consisting of a tone for 30 s (70 dB) and a foot shock (0.7 mA) during the last second of the tone followed by a 40 s inter-trial interval. Mice were subsequently returned to their home cage. Memory for the context was tested on day 2 by returning the mice to the chamber and allowing them to explore for 1 min whereafter freezing to the context was assessed for the remaining 4.5 min of the test. Here, the extent of freezing reflects the ability of the hippocampus to associate the aversive stimulus with the context. Memory for the tone (cue) was tested on day 3 in a disguised context (circular in shape, white color, different texture, and grape scent). Mice were allowed to explore the chamber for 2.5 min whereafter the tone was presented as day 1. Freezing was evaluated during inter-trial intervals. Here, the extent of freezing is considered reliant on amygdala. The time of freezing was monitored automatically by the StartFear system. Student’s unpaired *t*-tests were used to compare the percentage of time spent freezing between WT and *Sorcs2^−/−^* mice.

### Two-Bottle Choice Test

The mice (WT *n* = 8 and *Sorcs2^−/−^ n* = 8) were microchipped (Datamars slim transponder, Kruuse, Denmark) by anesthetizing the animal using isoflurane, making a small cut into the neck skin, inserting the microchip, and closing the wound with indermil xfine (Henkel, Germany).

The mice were group-housed in an automated water-intake monitoring system (HM-2 system, MBRose, Denmark). The mice were housed in the same groups as their home-cage (2–6 animals per cage). The mice were habituated to the HM-2 system cages and to the presence of two drinking bottles, which contained water, for 4 days. After acclimatization, both drinking bottles were shifted to containing a 10% ethanol solution (v/v). After 2 days, one of the bottles was shifted to containing water. The two bottles were switched every third day to reduce any confound by a side bias. The water and alcohol intake were monitored for 20 days including the 4 days for acclimatization and 2 days with forced alcohol intake. Differences in total water intake, total ethanol intake, forced water intake and forced ethanol intake between WT and *Sorcs2^−/−^* mice were determined using Mann–Whitney test, while differences in body weight and alcohol preference between WT and *Sorcs2^−/−^* mice were determined using mixed ANOVA with genotype and time as factors followed by Sidak’s multiple comparison test. For Figure 3B,C, area under curve was first calculated, where-after Mann–Whitney tests were conducted.

### Induction of Physical Dependence

The procedure was modified from previous described method (Pawlak et al., 2005). The mice were housed in groups in their normal housing cages (*n* = 2–3 for each genotype in control groups, *n* = 8 and *n* = 5 for ethanol groups of WT and *Sorcs2^−/−^*, respectively), and were gradually introduced to higher concentration of ethanol in their drinking water as follows: days 1–3, 2.3% ethanol; days 4–6. 4.7% ethanol; days 7–10, 7% ethanol, and days 11–14, 10%. Control groups received water without ethanol. The change of water containing increasing concentration of alcohol was done at 11 a.m. A pilot study together with the two-bottle choice test (see above) did not show any difference in intake of water containing alcohol between the two genotypes, when this was the only drinking option, therefore the intake of alcohol intake in water or alcohol containing water was not monitored in the given test. All groups had access to food *ad libitum*.

### Alcohol-Withdrawal and Assessment of Convulsion Severity

Alcohol-withdrawal was initiated on day 15 at 8:00 a.m. by removing the ethanol-containing water and replacing it with an ethanol-free water bottle.

Handling-induced withdrawal convulsion began with lifting the mouse from its cage and observing it for any convulsion. If no convulsion occurred when picked up, the mouse was gently spun clockwise and counterclockwise 360° using the thumb and forefinger for approximately 5 s. The mice were rated on a scale of 0–8: the mice were picked up by the tail and rated as follows: 0, no reaction when lifted by the tail, and no reaction after gentle 360° spin. Mouse is able to swing and grab its own tail; 1, no reaction when lifted by the tail, and no reaction after gentle 360° spin. Mouse is not able to swing and grab its own tail; 2, no reaction when lifted by the tail, and a slight jerkiness after gentle 360° spin; 3, no reaction when lifted by the tail, and a tonic convulsion after gentle 360° spin; 4, no reaction when lifted by the tail, and a clonic convulsion after gentle 360° spin; 5, tonic-clonic convulsion when lifted by the tail with onset within 2 s; 6, severe tonic-clonic convulsion when lifted by the tail with rapid onset and long duration; 7, spontaneous convulsion elicited by mild environmental stimuli (e.g., lifting the cage top); 8, death due to seizure. Data was analyzed using a Three-way ANOVA followed by *post hoc* Tukey’s multiple comparisons test or mixed ANOVA followed by *post hoc* Sidak’s multiple comparison test.

### Ethanol Assay on Blood Serum

Blood ethanol concentrations (BECs) were obtained 20 min after a 2.0 g/kg i.p. dose of ethanol (*n* = 4 WT mice and 9 *Sorcs2^−/−^* mice). Blood was drawn straight before sacrificing the mouse, and collected in microvette tubes lined with lithium heparin (SARSTEDT AG & Co, Germany). The BECs were measured using a commercial assay kit (Ethanol Assay kit, abcam65343) and was carried out following the instructions of the manufacturer. Absorbance was measured at OD 570 nm. Possible difference in BEC between WT and *Sorcs2^−/−^* mice was analyzed using a Student’s unpaired *t*-test.

### Statistics

For statistical comparisons, Prism 8.0 for Mac (GraphPad Software, La Jolla, CA, United States) was used. All data are presented as mean ± standard error of the mean (S.E.M.). Probability (*P*) values of less than 5% were considered statistically significant. For more details, see under each experiment in the method section.

## Results

### *Sorcs2*^−/−^ Mice Show No Alcohol-Induced Place Preference

**FIGURE 1:**
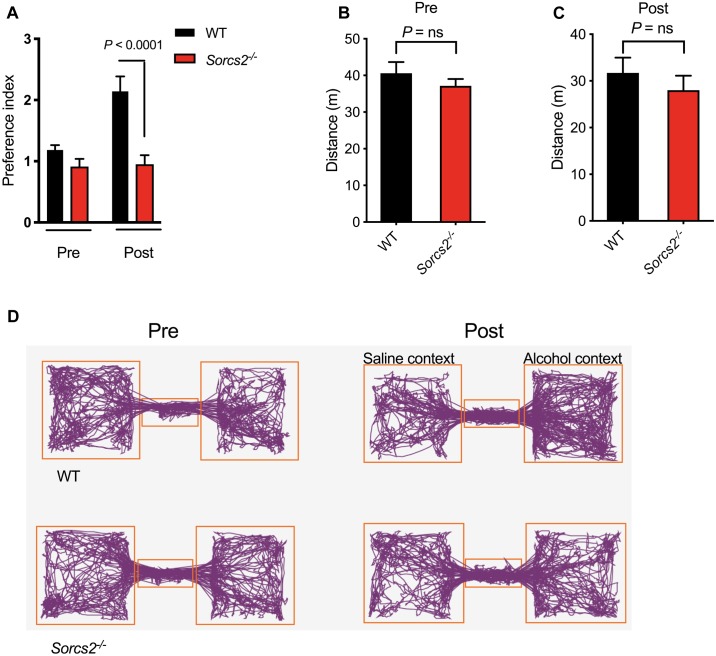
Lack of alcohol-induced place preference in *Sorcs2^−/−^* mice. **(A)** Preference index for WT and *Sorcs2^−/−^* for the alcohol conditioned context over the non-conditioned context, showing there was a significant difference between WT and *Sorcs2^−/−^* mice in acquiring alcohol-induced place preference (*P* < 0.0001). **(B,C)** Activity before (Pre) and after alcohol conditioning (Post), showed no significant difference in activity between the two genotypes (*P* = 0.63 for both). **(D)** Track plots illustrating activity in the three compartments of the preference box before and after alcohol conditioning (*n* = 6 for WT and *n* = 10 for *Sorcs2^−/−^*).

We investigated the reinforcing properties of alcohol in *Sorcs2^−/−^* mice and WT mice using the CPP paradigm. No mice displayed unconditioned preference or aversion, thus no mice were excluded for further testing (data not shown). In the post-conditioning phase, the mice were placed in the neutral compartment with free access to the two lateral compartments for 20 min. Mixed ANOVA analysis showed an interaction between genotype and conditioned preference for alcohol-paired chamber [*F*(1,15) = 7, *P* = 0.02]. A Sidak’s *post hoc* analysis showed that while there was no difference between the two genotypes in pre-condition (*P* = 0.60) a significant difference was observed during preference test (day 8, *P* < 0.0001). Thus, WT showed a preference index of 2.1 (*P* = 0.003) for the alcohol-associated compartment, while *Sorcs2^−/−^* mice did not acquire any preference for a particular compartment (preference index = 1.1, *P* = 0.63). Mixed ANOVA analysis showed no interaction between genotype and activity in pre- and post- conditioning phase [*F*(1,29) = 0.00, *P* = 0.97], and Sidak’s *post hoc* analysis showed that the activity of WT and *Sorcs2^−/−^* mice was the same in both pre-conditioning phase (*P* = 0.63) and post-conditioning phase (*P* = 0.63) (Figure 1).

### *Sorcs2^−/−^* Mice Have Intact Context- but Impaired Cue-Dependent Memory

**FIGURE 2:**
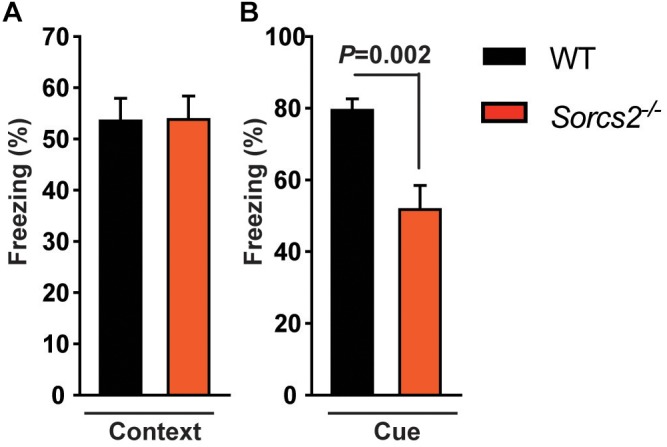
Intact context- but impaired cue-dependent memory in *Sorcs2^−/−^* mice. The time spent freezing in response to context **(A)** or cue **(B)** of WT and *Sorcs2^−/−^* mice in a context- and cue-dependent fear conditioning experiment, showing that *Sorcs2^−/−^* mice have intact contextual fear-learning (hippocampus) while cue-dependent memory (amygdala) was significantly reduced compared to WT mice (*P* = 0.002) (*n* = 21 for WT and *n* = 18 for *Sorcs2^−/−^*).

To rule out that the lack of place preference in *Sorcs2^−/−^* mice was due to deficient contextual memory, we performed a context- and cue-dependent fear conditioning experiment. We found that *Sorcs2^−/−^* mice displayed intact contextual fear-learning (hippocampus) while cue-dependent memory (amygdala) was significantly reduced compared to WT mice (*P* = 0.002, Student’s unpaired *t*-test) (Figure 2).

### *Sorcs2*^−/−^ Mice Show Decreased Alcohol Consumption in the Two-Bottle Choice Test

**FIGURE 3:**
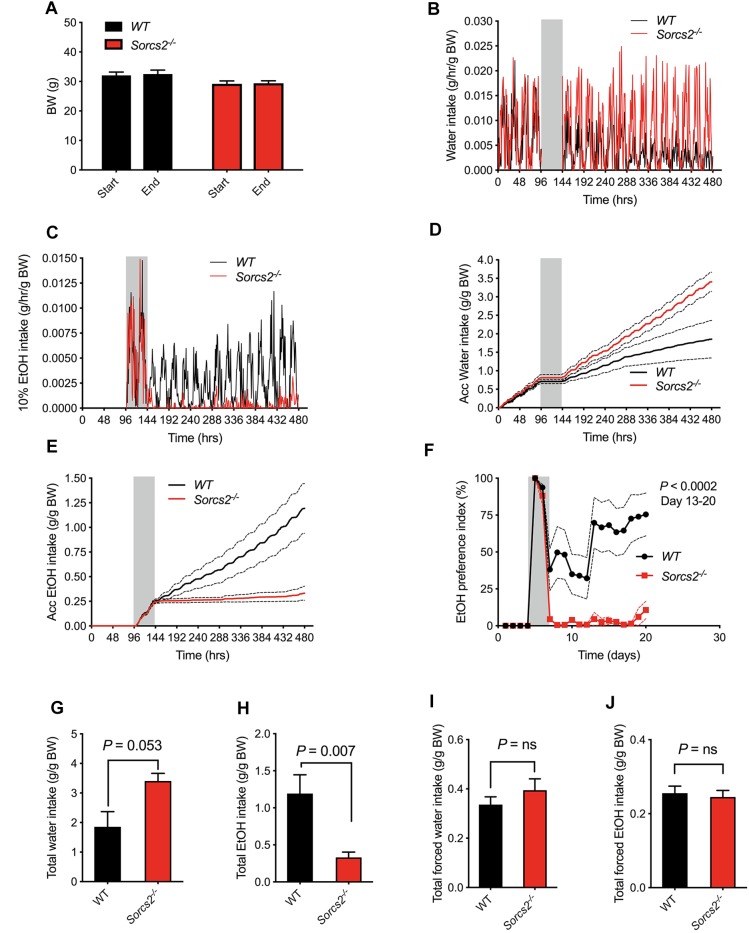
*Sorcs2^−/−^* mice show decreased voluntary alcohol consumption. **(A)** Bodyweight before and after experiment, showing no significant difference in bodyweight between WT and *Sorcs2^−/−^* mice (*P* > 0.9). **(B,D,G)** Intake of water during the experiment, showing there is a close to significant difference in intake of water between WT and *Sorcs2^−/−^* mice, where *Sorcs2^−/−^* mice had the highest intake (*P* = 0.0530). **(C,E,H)** Intake of 10% ethanol during the experiment showing that WT mice drank significantly more alcohol compared to WT mice (*P* = 0.007). **(F)** Preference index of ethanol over the course of the experiment, showing that WT had a significant higher preference for alcohol compared to *Sorcs2^−/−^* mice (*P* < 0.0002 for day 13–20). The gray bars (in panel **B–F**) indicate period with forced ethanol intake (no water available). There was no significant difference in intake of water when it was the only drinking option (*P* = 0. 46, panel **I**) or in forced ethanol intake (*P* = 0.81, panel **J**) (*n* = 8 mice per genotype). In panel **(B,C)**, mean without SEM are shown for sake of simplicity. Red lines are intake for *Sorcs2^−/−^* mice and black likes are intake for WT.

We first assessed whether there were any general difference in the drinking patterns between WT and *Sorcs2^−/−^* mice. In the beginning, when only water was available, no significant difference between the two genotypes was observed (Mann–Whitney test*, P* = 0.46). Next, the only drinking option was ethanol, and during this forced ethanol intake, there was no difference in intake of ethanol (Mann–Whitney test, *P* = 0.81). However, when the mice could choose between ethanol and water, *Sorcs2^−/−^* mice preferred water (preference index for alcohol = 11% on day 20), while WT preferred alcohol (preference index = 75% on day 20), Mixed ANOVA (*P* < 0.0002 on day 13–20). Overall, WT mice drank significantly more alcohol compared to *Sorcs2^−/−^* mice (*P* = 0.007, Mann–Whitney test), while *Sorcs2^−/−^* mice showed a tendency to drink more water compared to WT mice (*P* = 0.0530, Mann–Whitney test). There was no difference in weight between WT and *Sorcs2^−/−^* mice during the experiment [*F*(1,14) = 0.50, *P* = 0.49], mixed ANOVA, *P* = 0.14 in the start of the experiment and *P* = 0.10 at the end (Sidak’s multiple comparison test) (Figure 3).

### *Sorcs2*^−/−^ Mice Do Not Display Aversive Response to Alcohol Withdrawal

**FIGURE 4:**
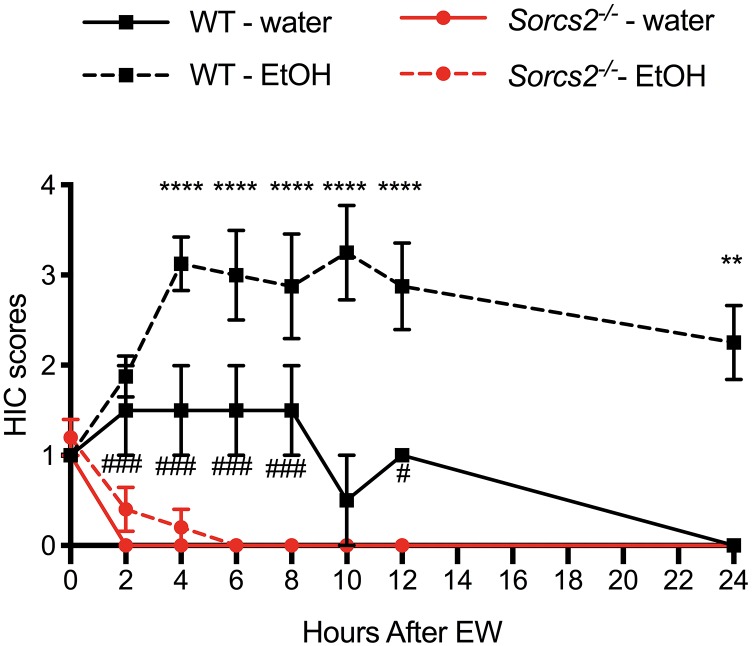
*Sorcs2^−/−^* mice do not display alcohol withdrawal symptoms. For 14 days, the only drinking source for WT and *Sorcs2^−/−^* mice was water containing alcohol, which increased in concentration over time. Control mice, continued to have normal tap water. On day 15, ethanol was withdrawn from the water. Hereafter, the mice were tested for handling-induced convulsion every 2 h after alcohol withdrawal. *Sorcs2^−/−^* mice seemed non-affected by the alcohol and its withdrawal. Accordingly, WT had significantly more severe convulsions (^∗∗∗∗^*P* < 0.0001 at 4, 6, 8, 10, 12 and ^∗∗^ *P* < 0.01 at 24 h after alcohol withdrawal, compared to *Sorcs2^−/−^* mice). Interestingly, higher level of convulsion was also observed in the WT control group compared to *Sorcs2^−/−^* control group (###*P* = 0.0001 and #*P* < 0.01) (*n* = 2–3 for each ethanol-naïve group and *n* = 5–8 for each ethanol-treated group). The results are expressed as mean ± SEM. The error bars in some data points are smaller than the symbols and therefore not visible.

To test if SorCS2 is involved in the severity of alcohol withdrawal symptoms, we measured the severity of alcohol-withdrawal symptoms in WT and *Sorcs2^−/−^* mice over time. For WT mice withdrawal symptoms peaked at 4 h, and remained stable for the next 6 h, whereafter they decreased. Contrary to WT mice *Sorcs2^−/−^* mice continued to have full control over their body and showed no sign of withdrawal symptoms. Although there was no significant interaction between Time × Treatment × Genotype [*F*(7,91) = 1.50, *P* = 0.17] there was a significant interaction between Time × Genotype [Three way ANOVA, *F*(7,91) = 3.6, *P* = 0.0016], and not quite significant interaction between Treatment × Genotype [*F*(1,13) = 4.10, *P* = 0.06]. Both genotype and treatment had main effects {[*F*(1,13) = 23, *P* = 0.0003] and [*F*(1,13) = 5.3, *P* = 0.03], respectively} while time had no main effect [*F*(7,77) = 1.1, *P* = 0.39]. *Post hoc* analysis showed a significant difference in convulsion severity between WT and *Sorcs2^−/−^* mice 4–24 h after withdrawal of alcohol (*P* < 0.01). Interestingly a significant difference between WT and *Sorcs2^−/−^* mice in response to handling-induced convulsion in the control groups was also observed, when comparing the two groups by themselves {mixed ANOVA [*F*(7,21) = 6.0, *P* = 0.0006], with both genotype and time having main effects [*F*(1,3) = 45, *P* = 0.007 and *F*(7,21) = 5.5, *P* = 0.001], respectively} (Figure 4).

### *Sorcs2*^−/−^ Mice Metabolize Alcohol Normally

**FIGURE 5:**
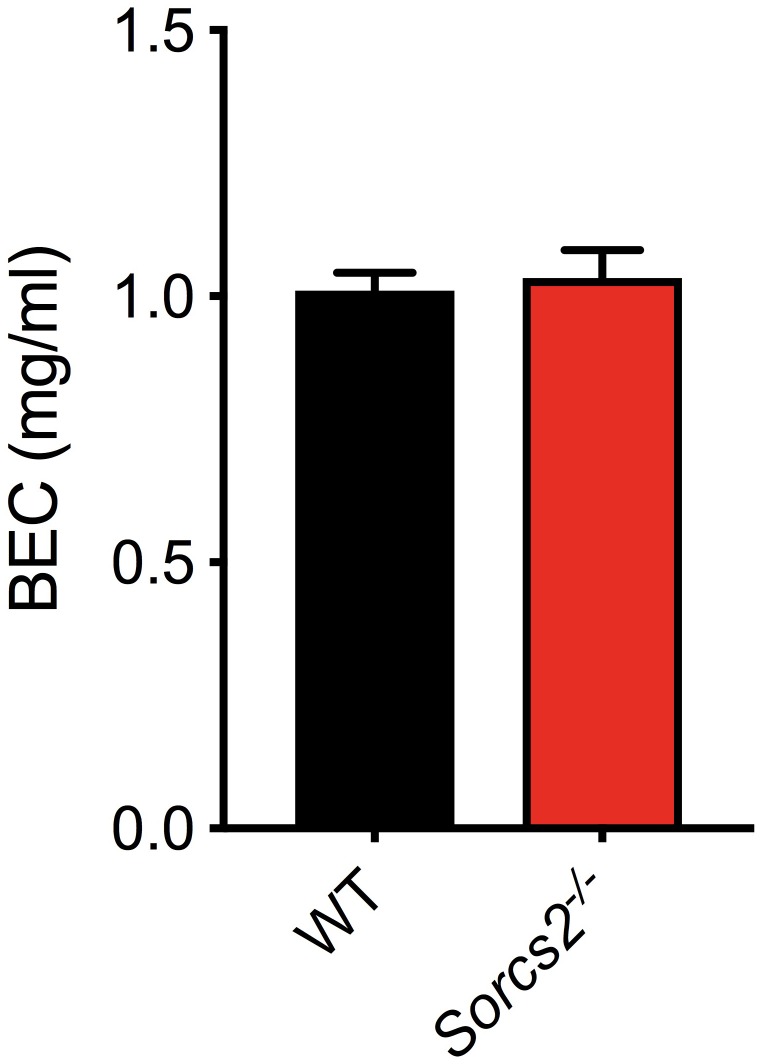
*Sorcs2^−/−^* mice metabolize ethanol normally. Blood ethanol concentrations BECs determined in WT and *SorCS2^−/−^* mice 20 min after a 2.0 g/kg ethanol i.p. injection. There were no significant differences in ethanol levels between the strains (*P* = 0.79) (*n* = 4 for WT and *n* = 9 for *SorCS2^−/−^* mice).

To ensure, that the observed differences in response to alcohol was not due to differences in metabolism of alcohol, blood from mice, which had been treated with alcohol (2.0 g/kg, i.p.), was collected. No significant difference in alcohol concentration was observed between WT and *Sorcs2^−/−^* mice (Student’s unpaired *t*-test, *P* = 0.79) (Figure 5).

## Discussion

We investigated alcohol-seeking behavior in mice lacking *Sorcs2* and found that, when compared to WT mice (I) *Sorcs2^−/−^* mice displayed complete lack of alcohol-induced place preference, (II) when given free choice between water and alcohol, *Sorcs2^−/−^* mice consumed less alcohol, and (III) *Sorcs2^−/−^* mice did not exhibit alcohol withdrawal symptoms. While WT acquired preference for alcohol, this was not the case for *Sorcs2^−/−^* mice. The absence of alcohol place preference observed in *Sorcs2^−/−^* mice does not mirror that of BDNF heterozygotes, BDNF Val/Met mice, or mice with inhibited TrkB activity, which all show both increased alcohol place preference and alcohol consumption (Hensler et al., 2003; McGough et al., 2004; Jeanblanc et al., 2006; Warnault et al., 2016), Instead, the *Sorcs2^−/−^* mouse phenotype likely reflects that elements of both TrkB and p75NTR activity are potentially perturbed in several neuronal systems, exemplified by deficient TrkB-dependent long term potentiation (LTP) and p75NTR-dependent long term depression (LTD) in the hippocampus of *Sorcs2^−/−^* mice (Glerup et al., 2016).

The NR2A subunit-containing NMDA receptors is also of importance for alcohol induced place preference (Boyce-Rustay and Holmes, 2006), and interestingly SorCS2 has been shown to regulate NMDAR trafficking by mediating surface trafficking of NR2A (Ma et al., 2017). Moreover, dysfunctions in NMDA receptor activity and plasticity have been observed in *Sorcs2^−/−^* mice (Glerup et al., 2016), which may also contribute to the altered acute and adapted response to alcohol observed here (Ron and Barak, 2016).

To ensure that the lack of development of alcohol place preference in *Sorcs2^−/−^* mice was not due to memory-impairments, the mice were subjected to context-cue dependent fear memory. Thus, as we found that mice lacking SorCS2 display intact context-dependent but impaired cue-dependent memory, this points toward a deficit in the amygdala. This phenotype is somewhat surprising considering that hippocampal LTP, which is completely absent in *Sorcs2^−/−^* mice, is believed to be instrumental for the formation of contextual memory, but not cue memory. However, a similar phenotype was reported for mice with conditional forebrain-specific deletion of TrkB (Minichiello et al., 1999). Thus, it is possible that SorCS2 and TrkB might also operate in concert to control memory processing in the amygdala. The amygdala, together with other parts of the dopaminergic system, is also part of the reward system, a system that plays a central role in the alcohol-induced responses in the brain (Soderpalm and Ericson, 2013). Thus, the fact that *Sorcs2^−/−^* mice have an altered dopaminergic system (Glerup et al., 2014b) could also contribute to the reduced preference for alcohol. Blocking the TrkB pathway by i.p. injections with the TrkB selective antagonist ANA-12 in mice reduces ethanol intake in a two-bottle choice paradigm (Leggio et al., 2014). Hence it is possible that altered TrkB signaling in *Sorcs2^−/−^* mice contributes to the reduced voluntary intake of ethanol observed in the two-bottle choice test. The observed alcohol preference observed in WT mice in the two-bottle choice experiment is consistent with previous findings (Belknap et al., 1993).

None of the Vps10p domain receptors have previously been implicated in the behavioral response to alcohol but the serum levels of the sortilin extracellular domain have been shown to correlate with alcohol consumption in humans (Buttenschon et al., 2015). Thus, it is tempting to speculate that abnormal expression of SorCS2 and possibly sortilin plays a role in the development of AUD in humans. Interestingly, the SORCS2 risk variant in subjects with serious alcohol withdrawals was identified to disrupt TF binding motifs within a stress hormone-responsive enhancer in human hippocampus, suggesting that regulation of SORCS2 expression is critical in the stress response associated with alcohol withdrawal. *Sorcs2^−/−^* mice also showed complete lack of handling-induced convulsion following alcohol withdrawal in treatment group but also in the control group, suggesting a general function of SorCS2 in the behavioral response to stress.

## Conclusion

In this study we show that lack of SorCS2 results in altered acute and adapted behavioral response to alcohol in mice. *Sorcs2^−/−^* mice lack alcohol place preference at a dose that induces place preference in WT mice, and display decreased voluntary alcohol consumption. Moreover, unlike WT mice, *Sorcs2^−/−^* mice do not develop alcohol withdrawal symptoms. Future studies should address the individual contributions of SorCS2/TrkB and SorCS2/p75NTR complexes to alcohol seeking behavior.

## Ethics Statement

All experiments were approved by the Danish Animal Experiments Inspectorate under the Ministry of Justice (Permit 2011/561-119, 2016-15-0201-01127 and 2017-15-0201-01192) and carried out according to institutional and national guidelines.

## Author Contributions

DO, JL, SØ, and SG designed the research. DO, MatK, MadK, JL, and SM performed the experiments. DO, MatK, MadK, JL, and SG analyzed the data. AN contributed with expertise in mouse models and with analytical tools. DO, JL, SØ, and SG drafted the manuscript.

## Conflict of Interest Statement

The authors declare that the research was conducted in the absence of any commercial or financial relationships that could be construed as a potential conflict of interest.
